# Glucocorticoid Functional Reserve in Full-Spectrum Intensity of Primary Hypothyroidism

**DOI:** 10.1155/2014/313519

**Published:** 2014-08-07

**Authors:** René Rodríguez-Gutiérrez, Camilo González-Velázquez, Gerardo González-Saldívar, Jesús Zacarías Villarreal-Pérez, José Gerardo González-González

**Affiliations:** ^1^Endocrinology Division, Internal Medicine Department, University Hospital “Dr. José E. González”, Facultad de Medicina, Universidad Autónoma de Nuevo León, Avenida Madero y Avenida Gonzalitos s/n, Colonia Mitras Centro, 64460 Monterrey, NL, Mexico; ^2^Internal Medicine Department, University Hospital “Dr. José E. González”, Facultad de Medicina, Universidad Autónoma de Nuevo León, Avenida Madero y Avenida Gonzalitos s/n, Colonia Mitras Centro, 64460 Monterrey, NL, Mexico

## Abstract

Adrenal function might be impaired in patients with primary hypothyroidism. The objective was to evaluate adrenocortical function using the low-dose cosyntropin test in patients with primary hypothyroidism. Consequently a prospective, longitudinal, controlled study of sixty adult patients with primary hypothyroidism was carried out. Patients* naïve* to L-T4 treatment were compared with control participants. A low-dose cosyntropin test was done before and after L-T4 therapy. Thirty and sixty minutes after the low-dose cosyntropin, the mean cortisol values were lower in the cases group (612.6 ± 133.1 and 603.4 ± 153.7 nmol/L) when compared to the control group (677.0 ± 82.4 and 669.9 ± 83.7 nmol/L) (*P* = 0.001 and 0.003), respectively. After L-T4 therapy, this difference was lost. Four out of 60 cases (6.7%) taking a cortisol cutoff value of 500 mmol/L and 11 out of 60 (18.3%) having 550 mmol/L as the cutoff had adrenal insufficiency before L-T4 therapy. After L-T4 therapy, 50% and 81% of the cases had normal cortisol response. In conclusion, patients with different degrees of intensity of primary hypothyroidism had improved cortisol response after reaching euthyroidism. The incidence of adrenal insufficiency was 6.7–18.3% and more than 50% of the cases had a normal cortisol response after L-T4 therapy.

## 1. Introduction

Thyroid gland dysfunction is a highly prevalent disorder in day-to-day clinical practice. In fact, one of the most common reasons for primary care consult is primary hypothyroidism. Recent studies have found up to 25% prevalence in women aged 60 and over [1–5]. The majority of these cases are either subclinical or minimally symptomatic primary hypothyroidism [6]. The current widespread availability of thyroid function tests has allowed frequent and early detection of this disorder despite a low-index of clinical suspicion. In consequence, severe primary hypothyroidism is now seldom diagnosed. It is common practice to initiate levothyroxine (L-T4) replacement together with glucocorticoids in extreme conditions, such as myxedema coma [7]. Once patients are clinically stable, glucocorticoids are discontinued. This therapeutic approach is based on small series of cases that have described transient adrenocortical impairment due to thyroid dysfunction and vice versa [8–15]. Most of the evidence, however, has methodological design problems that make the clinical implications of these conclusions uncertain.

Some nondistinctive clinical manifestations of hypothyroidism and adrenocortical insufficiency may overlap, particularlly in the musculoskeletal and neurological systems [16]. After starting L-T4 therapy in patients with hypothyroidism, some of these symptoms may worsen due to myxedema resolution or temporary adrenocortical dysfunction [17]. An equilibrium between low cortisol secretion and its decreased metabolism could lead to apparently normal basal serum and urine cortisol values [8, 11]. A transitory low glucocorticoid production has been identified in cases with severe hypothyroidism but assessment of glucocorticoid functional reserve in compensated and mild to moderate cases of primary hypothyroidism has not been well elucidated. Furthermore, the higher sensitivity of the low-dose cosyntropin test has not been explored before L-T4 therapy and at follow-up in these patients [18].

Accordingly, a prospective, longitudinal, and controlled study was carried out to evaluate adrenocortical glucocorticoid function using the low-dose cosyntropin test, in patients with subclinical, mild-moderate, and severe primary hypothyroidism. The primary endpoint was to contrast the cortisol response to low-dose cosyntropin before L-T4 replacement and after normalization of thyroid function tests, between groups and in each category and, furthermore, to compare the cortisol values before and after L-T4 replacement in cases versus their controls. A secondary endpoint was to identify the prevalence of transient or permanent adrenocortical insufficiency.

## 2. Subjects and Methods

### 2.1. Subjects

After obtaining approval from the Research and Ethics Committee and signed informed consent from each subject, we began the study. Patients were recruited through the internal medicine, primary care, and endocrinology clinics of the “Dr. José E. González” University Hospital. A total of 60 men or women, aged 18 to 70 years, with a diagnosis of primary hypothyroidism and* naïve* to L-T4 treatment, were included. Primary hypothyroidism was documented by the results of the total and free thyroxin levels (TT4 and FT4, resp.), thyrotropin (TSH) values, and positive antibodies to thyroid peroxidase. Cases were classified into three groups: subclinical primary hypothyroidism ((group #1) (*n* = 30) (normal TT4 and FT4 values with an elevated TSH result but below 10 *μ*U/mL)), mild/moderated hypothyroidism ((group #2) (*n* = 15) (low TT4 and FT4 associated with a TSH value between 20 and 40 *μ*U/mL + mild symptoms)), and severe hypothyroidism ((group #3) (*n* = 15) (very low TT4 and FT4 associated with a TSH value > 100 *μ*U/mL + intense symptoms)). A complete clinical history and physical examination were performed in all participants. We excluded individuals with other endocrinopathies, glucocorticoid use in the last 24 months, and current use of oral/nonoral contraceptives or drugs that may interfere with cortisol metabolism, transport, or the hypothalamo-pituitary-adrenal axis. An age and gender matched control group (*n* = 60) of healthy subjects was recruited from the primary care clinic.

### 2.2. Study Protocol

To perform a low-dose cosyntropin test, a solution was prepared from serial dilutions in normal saline of one vial of 250 *μ*g synthetic ACTH (Cortrosyn, Organon Inc., West Orange, NJ, USA), first to 50 *μ*g/mL, then to 5 *μ*g/mL, and finally to the 1 *μ*g/mL concentration. The vials were maintained refrigerated at 4°C for a 3-month period. All participants, cases and controls, underwent a 1 *μ*g cosyntropin dose test. After overnight fasting, between 8 and 9 h, an intravenous plastic catheter was inserted into a forearm vein and a baseline blood sample was drawn for measurement of basal cortisol one minute before each test. Subsequently, 1 *μ*g ACTH was injected as a bolus through the catheter and flushed afterwards with 10 mL of normal saline. Blood samples for serum cortisol were taken at 30 and 60 min. All samples were centrifuged, separated immediately, and frozen at −20°C. A serum cortisol value equal to or greater than 500 nmol/L at 30 or 60 min after stimulation was defined as a normal glucocorticoid response [18–20]. Five hundred and fifty nmol/L was an alternate cutoff value that was also examined [20, 21].

A low-dose cosyntropin test was carried out at baseline in all cases (before starting L-T4 therapy) and repeated 16 weeks later during follow-up, once thyroid function tests (TT4, FT4, and TSH) were documented to be within normal values. All cases received L-T4 replacement therapy as recommended [12]. In the control group a low-dose cosyntropin test was performed only at baseline.

### 2.3. Measurements

Thyroid function testing included measurement of TT4, FT4, and TSH. Total and free thyroxin were measured using a commercial kit for electrochemiluminescence (Modular Analytics E170, Roche/Hitachi Diagnostics, Mannheim, Germany). Intra-assay variation coefficients were 3.7, 3.4, and 4.2% and 2.7, 2.6, and 3.6% for low, medium, and high TT4 and FT4 values, respectively. TSH was measured using a commercial electrochemiluminescence kit (Modular Analytics E170, Roche/Hitachi Diagnostics, Mannheim, Germany). Intra-assay variation coefficients were 7.2, 3.2, and 3.3% for low, medium, and high TSH values, respectively. Serum cortisol was measured using a commercial RIA kit (Elecsys 2010, Roche/Hitachi Diagnostics, Mannheim, Germany). Intra-assay variation coefficients were 1.6, 1.5, and 1.6% for low, medium, and high cortisol values, respectively. All samples were assessed twice.

### 2.4. Statistical Analysis

All results are reported as mean ± standard deviation unless otherwise stated. A *P* value equal to or less than 0.05 was considered statistically significant. Descriptive statistical analysis was used for quantitative variables, measures of central tendency, and dispersion. In the case of qualitative variables, frequencies were obtained. To compare differences between basal and follow-up cortisol responses within each patient group and between patients and controls, paired and unpaired Student's *t* tests were used, respectively. The statistical analysis was performed with IBM SPSS Statistics 20.0 (SPSS Inc., Armonk, NY).

## 3. Results

### 3.1. Study Population

The demographic characteristics of the study participants are shown in Table 1. There were 30, 15, and 15 participants in groups 1, 2, and 3, respectively. The majority of cases were between 18 and 60 years of age and, as expected, most were women (75%). There was no statistically significant difference in age between cases and age-gender matched controls (*P* = 0.83). All cases and controls completed the study.

### 3.2. Assessment before Levothyroxine Therapy

#### 3.2.1. Cases versus Controls: Comparison of Cortisol Values of All Cases and Controls

Mean baseline (0 min) cortisol value between all cases (357.3 ± 144.8 nmol/L) and controls (369.2 ± 91.4 nmol/L) was not statistically significant (*P* = 0.58). See Table 2. Thirty and sixty minutes after the cosyntropin test the mean cortisol value was statistically lower in the cases group (612.6 ± 133.1 and 603.4 ± 153.7 nmol/L) when compared to the control group (677.0 ± 82.4 and 669.9 ± 83.7 nmol/L) (*P* = 0.001 and 0.003), respectively.

#### 3.2.2. Cases versus Controls: Comparison of Cortisol Values by Groups (Subclinical, Mild/Moderate, and Severe)

Comparison of the cortisol values (baseline, 30 and 60 minutes) between group 1 and their controls was not statistically different. When group 2 was compared with controls a lower mean cortisol value at 60-minute time (598.9 ± 135.0 nmol/L, 686.5 ± 66.3 nmol/L, resp.) was observed in the case group (*P* = 0.03). Group 3 had a lower cortisol value at 30 minutes (540.9 ± 178.7 nmol/L) when compared with controls (687.9 ± 61.6 nmol/L). All other cortisol values, even though lower in cases than in controls, were not statistically different. See Table 2.

#### 3.2.3. Comparison of Cortisol Values between Cases Groups (Subclinical, Mild/Moderate, and Severe)

Mean baseline cortisol values were not different between all three groups of cases (384.6 ± 152.2 SD, 346.9 ± 148.5 SD, and 349.0 ± 157.5 nmol/L). Groups 1 versus 2, 2 versus 3, and 1 versus 3 resulted in a *P* value of 0.43, 0.48, and 0.46, respectively. At 30-minute time there was a statistical difference between groups 1 versus 3 and 2 versus 3 (*P* = 0.01 and 0.04, resp.) due to a lower mean cortisol value in group 3 participants. This significant difference disappeared at 60-minute time comparison between groups 1 versus 2, 1 versus 3, and 2 versus 3 (*P* = 0.12, 0.83, and 0.11, resp.). See Table 3.

### 3.3. Assessment after Levothyroxine Therapy

#### 3.3.1. Cases versus Controls: Comparison of Cortisol Values of All Cases and Controls

Once levothyroxine replacement reached normalization of the thyrotropin values in all cases, comparison of the mean serum cortisol values at baseline, 30, and 60 minutes after stimulation against the control group did not show any difference. See Table 3.

#### 3.3.2. Cases versus Controls: Comparison of Cortisol Values by Groups (Subclinical, Mild/Moderate, and Severe)

Mean serum cortisol values at baseline, 30, and 60 minutes after cosyntropin stimulation did not reach statistical differences in groups 1, 2, and 3 when compared with their controls. See Table 4.

#### 3.3.3. Comparison of Cortisol Values between Cases Groups (Subclinical, Mild/Moderate, and Severe)

Mean cortisol values were different between group 2 and group 3 at baselines 302.3 ± 87.4 and 442.5 ± 198.4 nmol/L; *P* = 0.009. The rest of the comparisons between groups at baseline and at 30 and 60 minutes were no different. See Table 3.

### 3.4. Longitudinal Comparisons of Cortisol Values (before versus after Levothyroxine Replacement)

#### 3.4.1. Longitudinal Comparison of Cortisol Values in the Case Group

When cases were compared before and after L-T4 therapy, mean serum cortisol values were lower before thyroid hormone normalization. Nevertheless, statistical significance was found only at 30-minute time (612.6 ± 133.1 versus 653.2 ± 138.3 nmol/L). *P* = 0.04. See Table 4.

#### 3.4.2. Longitudinal Comparison of Cortisol Values by Groups (Subclinical, Mild/Moderate, and Severe)

The mean cortisol values at baseline and after stimulation with cosyntropin when compared before and after levothyroxine in group 1 showed lower values before L-T4 replacement but statistical significance was reached only at 60-minute time (*P* = 0.03). In group 2 there was no statistically significant difference between mean cortisol values before and after L-T4 therapy. In group 3 the mean cortisol values at baseline and 30-minute time were statistically lower before L-T4 (*P* = 0.02 and 0.01, resp.). See Table 5.

### 3.5. Glucocorticoid Functional Insufficiency before and after L-T4 Therapy

Taking 500 nmol/L or more as the normal cortisol response at 30 or 60 minutes after cosyntropin stimulation, 4 out of 60 cases (6.7%) had insufficient glucocorticoid reserve before L-T4 treatment, one of them in group 1 and three in group 3. After L-T4 therapy 2 out of 4 cases reached a normal cortisol response and the other two cases (both in group 3) stayed with glucocorticoid insufficiency. They had an elevated plasma adrenocorticotropic level (291 and 443 pg/mL; normal range: less than 46 pg/mL) and glucocorticoid replacement therapy was initiated. Adrenal cortex antibodies were not carried out. When a cortisol value of 550 nmol/L or more was taken as the normal cortisol response, 11 out of 60 cases (18.3%) had an abnormal cortisol response to cosyntropin at 30 and 60 min, six of them in group 3, three in group 2, and two in group 1. After L-T4 replacement 9 out of 11 cases (81.8%) showed a normal cortisol response. The two persistent abnormal cases were in group 3. In the control group, all of the participants had an adequate cortisol response after cosyntropin stimulation at 30 or 60 minutes with both 500 nmol/L and 550 nmol/L cutoff values.

## 4. Discussion

In this prospective study of patients with different degrees of intensity of primary hypothyroidism we found with the low-dose cosyntropin test that their serum cortisol response before L-T4 replacement therapy was significantly lower than controls. After thyroid hormone level normalization, an increase in their cortisol response was observed and this difference was lost. This change was primarily due to an increase in the cortisol response in the severe hypothyroidism group. The analysis by group of cases (subclinical, mild/moderated, and severe) after L-T4 therapy showed a modest but significant increase in cortisol response at 0 and 30 minutes in the severe hypothyroidism group and at 60 minutes in the subclinical hypothyroidism group. Remarkably, 4 out of 60 cases (6.7%) with 500 nmol/L as the cortisol cutoff value and 11 out of 60 cases (18.3%) with the 550 nmol/L as a cutoff had an abnormal cortisol response (adrenal insufficiency) that reverted after L-T4 therapy in 2 (50%) and 9 cases (81.8%), respectively, without any other treatment.

It has been generally accepted that adrenal function might be impaired in patients with primary hypothyroidism. In 1957, Peterson in seven patients with severe hypothyroidism described that cortisol secretion and synthesis were reduced, primarily due to an impairment of cortisol metabolism by the liver. After L-T4 therapy was initiated cortisol production returned to normal. However, not every patient was evaluated after L-T4 therapy and adrenal function was not assessed using a stimulation test [8]. Later, in patients with primary hypothyroidism, Macgregor and Lessof et al. proposed an insufficient adrenal response to ACTH stimulation and an impairment of pituitary function, respectively [9, 10]. On the other hand, a normal HPA axis in patients with primary hypothyroidism has also been reported [11, 14]. In 1970, in a study by Harvard et al., in seven patients with primary hypothyroidism and in three with secondary hypothyroidism, adrenal stimulation with a standard ACTH test showed a normal cortisol response at 30 and 60 min in all patients before L-T4 therapy. This remained unchanged after achieving euthyroidism. Nevertheless, in that same study insulin-induced hypoglycemia produced a significant increase in cortisol response after L-T4 treatment [11]. Recently, Ünlühizarci et al. evaluated the HPA axis comparing the low-dose versus standard dose cosyntropin test before L-T4 therapy in 14 patients with severe primary hypothyroidism (TSH > 100 *μ*U/mL) [14]. The cortisol response in cases, although higher with the standard cosyntropin dose, was not statistically different. Interestingly, in these patients, cortisol response with either test was not statistically different from the cortisol response of the control group, suggesting that adrenal function was not impaired in primary hypothyroidism. Assessment of the cortisol response after L-T4 therapy was not carried out [14]. In the present study, we describe the cortisol response using the low-dose cosyntropin test in full-spectrum primary hypothyroidism patients (subclinical, mild/moderate, and severe) before and after normalization of thyroid function tests. When these patients were compared with age- and gender-matched controls an improved cortisol response was found, and so was reversal of adrenocortical insufficiency in over 50% of the cases after L-T4 therapy.

The exact site and how the HPA axis is impaired by hypothyroidism are still not well elucidated. It has been described that thyroidectomy decreases plasma ACTH levels (even though the pituitary content of ACTH might be increased) due to a reduction in corticotropin releasing hormone (CRH) gene expression in the hypothalamus [22, 23]. Additionally, in hypothyroid-induced rats, a reduction in ACTH and a significant decrease in adrenal weight along with an increase of ACTH to CRH stimulation suggest a central adrenal insufficiency mediated by low thyroid hormone levels that has been described to be more pronounced as the duration and intensity of thyroid dysfunction increase. This situation has been explained by decreased CRH mRNA expression in the hypothalamic paraventricular nucleus. At the adrenal level, in long-standing hypothyroidism, a significant reduction in cortisol secretion after ACTH has been documented in primary adrenal cell culture [15]. This finding was also supported by Tohei et al. who suggest that hypothyroidism directly causes adrenal dysfunction and that hypersecretion of CRH and arginine vasopressin is due to a reduction in the negative feedback effect of glucocorticoids [23]. In our study, ACTH levels were mildly elevated in hypothyroid patients that had an inadequate cortisol response to cosyntropin before L-T4 therapy. Remarkably, adrenal function was completely restored after reaching a euthyroid state in 9 out of 11 patients using the 550 nmol/L cortisol cutoff value. These results suggest a direct (adrenal level) and transitory rather than permanent adrenal dysfunction provoked by low concentration of thyroid hormone levels. As previously reported in rats, most of our cases were from the severe hypothyroidism group. However, between 25–50% of patients in the mild/moderate groups also had an initial abnormal cortisol response that was normalized in all cases after successful L-T4 therapy.

Interestingly, cortisol response to cosyntropin was not impaired in all hypothyroid patients. This suggests that even though low serum thyroid hormones play an important role in adrenal dysfunction, there are other associated factors that still have to be recognized. Two of our cases remained with adrenal insufficiency despite adequate L-T4 therapy. Both were from the severe hypothyroidism group and even though the possibility of a permanent adrenal dysfunction due to low thyroid hormone levels is plausible, it is important to remember the link between hypothyroidism and adrenal insufficiency. It is likely that these cases could have an underlying autoimmune etiology.

It is worthy to mention that even though cortisol levels improved after L-T4 therapy mean basal and stimulated cortisol levels in the case group were within normal range in all subgroups. When this data is seen as a whole, it suggests that in primary hypothyroidism there is an impaired HPA axis that improves with L-T4 therapy. Nonetheless, this condition will probably have a discrete, if at all, clinical significance as cortisol levels at baseline and after cosyntropin stimulation before and after L-T4 therapy remained within normal range. However, when each case is viewed separately, 4 out of 60 cases (6.7%) with a cortisol cutoff value of 500 mmol/L and 11 out of 60 (18.3%) with a cutoff of 550 mmol/L had adrenal insufficiency before L-T4 therapy. Remarkably, 50% and 81% of the cases had a normal cortisol response after L-T4 therapy without any other treatment. Most of the cases (>50%) were in the severe hypothyroidism group, suggesting, as previously described in rats, that a prolonged state of hypothyroidism might deeply impair cortisol secretion [16]. Nevertheless, in our study, 25–50% of the cases of adrenal insufficiency were in the subclinical-mild/moderate hypothyroidism groups. This feature has important clinical implications since we confirm the necessity of assessing adrenal function with the low cosyntropin test in all severe hypothyroidism cases. Due to the high incidence of compensated/mild hypothyroidism we have a strong clinical suspicion of the coexistence of transient adrenocortical insufficiency. In these cases, a low-dose ACTH test could be performed. A clinical scenario could be in situations of acute stress which occur while a euthyroid state is still not achieved and that, if not treated, could otherwise potentially lead to an acute adrenal crisis. A cortisol value at 30 or 60 minutes after cosyntropin stimulation greater than 550 nmol/L in all subjects in the control group strongly supports the important role of thyroid hormones in HPA axis dysfunction and suggests that 550 nmol/L might be a better serum cortisol cutoff for the diagnosis of an insufficient adrenocortical response.

A direct TSH elevation secondary to adrenal insufficiency with reversal of thyroid function after glucocorticoid replacement has been described [12, 24–26]. In 1980, Topliss et al. reported that six out of ten patients with primary adrenal insufficiency had a mild/moderate increase in TSH levels that reversed to normal values after glucocorticoid therapy. Interestingly, TSH oscillations occurred without a change in thyroid hormone levels. In all patients rT_3_ levels were the same before and after treatment suggesting that TSH could be increased as a direct effect of adrenal deficiency without thyroid dysfunction. Thyroid antibodies in all six patients were negative. The patients in whom TSH remained high had positive thyroid antibodies and higher TSH levels. The proposed mechanism was that low cortisol levels cause a rise in pro-TRH mRNA in the hypothalamic paraventricular nucleus with a consequent increase of TSH levels [12]. Barnett et al. reported similar findings in five patients with Addison's disease and TSH elevation. In all of these cases anti-thyroglobulin or thyroid peroxidase antibodies were positive and only one case remained euthyroid without thyroid replacement after glucocorticoid therapy. Remarkably, six out of eleven patients with Addison's disease had normal thyroid function tests [25]. In our study, all cases had documented low TT4 and FT4 with positive thyroid antibodies. Only two of the eleven cases with inadequate adrenal response did not recover after L-T4 therapy, both cases were from the severe hypothyroidism group. In all cases of mild/moderate hypothyroidism, cortisol response to cosyntropin became normal after L-T4 therapy. This suggests that even though TSH levels could have increased because of glucocorticoid deficiency, these were only exacerbated by this phenomenon rather than being the primary cause.

It has been debated for a long time which test (low or standard dose) cortisol stimulation has better performance. Based on the concept that 250 *μ*g of cosyntropin is a pharmacologic rather that a physiologic stimulus that may stimulate partial atrophied or mildly dysfunctional adrenal glands, multiple studies have demonstrated that the low-dose cosyntropin test has better or equal results than the standard test. Particularly, in the setting of chronic partial pituitary ACTH deficiency and recent onset ACTH deficiency, low-dose stimulation has demonstrated a better performance [18, 19, 27–33]. This is a strength of our study since most of the patients with abnormal response to low-dose cosyntropin (1 *μ*g) had a near-normal/partial abnormal cortisol response that could have been otherwise overestimated if we had used the standard 250 *μ*g cosyntropin test.

In conclusion, this prospective study of patients with different severities of primary hypothyroidism showed, using the low-dose cosyntropin test, an improved cortisol response after normalization of thyroid hormone levels. The mechanism of impaired cortisol secretion in cases with hypothyroidism still has to be elucidated but our data support an adrenal rather than a pituitary affection. The incidence of adrenal insufficiency was 6.7–18.3% and more than 50% of the cases had a normal cortisol response after L-T4 therapy. Remarkably, 25–50% of the cases were in the subclinical/mild/moderate hypothyroidism groups. This finding could have important clinical implications especially in the setting of acute stress situations occurring during the period while a euthyroid state is still not achieved.

## Figures and Tables

**Table 1:** Demographic characteristics of the participants.

	Cases			Cases	Controls
	Group 1 (*n* = 30)	Group 2 (*n* = 15)	Group 3 (*n* = 15)	Total (*n* = 60)	Total (*n* = 60)
Age—yr.	42.0 ± 12.6	43.8 ± 12.8	41.2 ± 12.7	42.3 ± 12.8	41.8 ± 12.0
Female no. (%)	24 (40.0)	11 (18.3)	10 (16.6)	45 (75.0)	45 (75.0)
Male no. (%)	6 (10.0)	4 (6.6)	5 (8.3)	15 (25.0)	15 (25.0)
18–40 yrs no. (%)	13 (21.7)	7 (11.6)	6 (10.0)	26 (43.3)	26 (43.3)
41–60 yrs no. (%)	14 (23.4)	5 (8.3)	8 (13.3)	27 (45.0)	27 (45)
>60 yrs no. (%)	3 (5.0)	3 (5.0)	1 (1.6)	7 (11.6)	7 (11.6)

Group 1 denotes subclinical hypothyroidsm; Group 2: mild/moderate hypothyroidsm; Group 3: severe hypothyroidsm; Yrs: years; %: percentage. Plus-minus values are means ± standard deviation.

**Table 2:** Comparative cortisol values to cosyntropin before L-T4 replacement therapy between cases and their age-matched controls (nmol/L).

	Cases (*n* = 60)	Controls (*n* = 60)	*P* value
All (*n* = 60)			
0 min	357.3 ± 144.8	369.2 ± 91.4	0.58
30 min	612.6 ± 133.1	677.0 ± 82.4	0.001∗
60 min	603.4 ± 153.7	669.9 ± 83.7	0.003∗
Group 1 (*n* = 30)			
0 min	384.6 ± 152.2	383.3 ± 110.5	0.96
30 min	652.5 ± 101.3	673.2.0 ± 79.1	0.38
60 min	618.3 ± 177.0	679.1 ± 95.6	0.10
Group 2 (*n* = 15)			
0 min	346.9 ± 148.5	348.6 ± 85.9	0.97
30 min	636.5 ± 112.8	663.7 ± 108.1	0.36
60 min	598.9 ± 135.0	686.5 ± 66.3	0.03∗
Group 3 (*n* = 15)			
0 min	348.9 ± 157.5	361.7 ± 40.4	0.76
30 min	540.9 ± 178.7	687.9 ± 61.6	0.005∗
60 min	611.7 ± 115.6	634.8 ± 66.32	0.50

All values given in nmol/L to convert to mg/dL are divided by 27.59.

∗Significant at *P* of ≤0.05.

**Table 3:** Glucocorticoid functional assessment in the cases by groups (nmol/L).

	Group 1	1 versus 2	Group 2	2 versus 3	Group 3	1 versus 3
		*P*		*P*		*P*
0 min^†^	384.6 ± 152.2	0.43	346.9 ± 148.5	0.48	349.0 ± 157.5	0.46
0 min^‡^	391.9 ± 176.3	0.07	302.3 ± 87.4	0.009∗	442.5 ± 198.4	0.38
30 min^†^	652.5 ± 101.3	0.66	636.5 ± 112.8	0.04∗	540.9 ± 178.7	0.01∗
30 min^‡^	678.3 ± 155.5	0.41	640.7 ± 119.9	0.34	662.2 ± 167.9	0.75
60 min^†^	618.3 ± 177.0	0.71	598.9 ± 135.0	0.39	611.7 ± 115.6	0.89
60 min^‡^	698.4 ± 148.0	0.12	629.9 ± 94.9	0.83	619.8 ± 166.6	0.11

All values given in nmol/L to convert to mg/dL are divided by 27.59.

∗Significant at *P* ≤ 0.05, paired *t*-test.

^†^Cases documented with primary hypothyroidism at baseline.

^‡^Values obtained in cases after LT-4 tratment.

**Table 4:** Comparative cortisol values to cosyntropin after LT-4 replacement therapy between cases and their age-matched controls (nmol/L).

	Cases (*n* = 60)	Controls (*n* = 60)	*P* value
All (*n* = 60)			
0 min	367.5 ± 149.1	369.2 ± 91.4	0.93
30 min	653.2 ± 138.3	677.0 ± 82.4	0.25
60 min	661.6 ± 144.4	669.9 ± 83.7	0.89
Group 1 (*n* = 30)			
0 min	391.9 ± 176.3	383.3 ± 110.5	0.82
30 min	678.3 ± 155.5	673.2.0 ± 79.1	0.87
60 min	698.4 ± 148.0	679.1 ± 95.6	0.54
Group 2 (*n* = 15)			
0 min	302.3 ± 87.4	348.6 ± 85.9	0.15
30 min	640.7 ± 119.9	663.7 ± 108.1	0.43
60 min	629.9 ± 94.9	686.5 ± 66.3	0.07
Group 3 (*n* = 15)			
0 min	442.5 ± 198.4	361.7 ± 40.4	0.13
30 min	662.1 ± 167.8	687.9 ± 61.6	0.58
60 min	619.7 ± 166.3	634.8 ± 66.32	0.74

All values given in nmol/L to convert to mg/dL are divided by 27.59.

**Table 5:** Comparative cortisol values to cosyntropin after LT-4 replacement therapy between cases and their age-matched controls (nmol/L).

	Before L-T4 (*n* = 60)	After L-T4 (*n* = 60)	*P*
All (*n* = 60)			
0 min	357.3 ± 144.8	367.5 ± 149.1	0.70
30 min	612.6 ± 133.1	653.2 ± 138.3	0.04∗
60 min	603.4 ± 153.7	661.6 ± 144.4	0.06
Group 1 (*n* = 30)			
0 min	384.6 ± 152.2	391.9 ± 176.3	0.86
30 min	652.5 ± 101.3	678.3 ± 155.5	0.44
60 min	618.3 ± 177.0	698.4 ± 148.0	0.03∗
Group 2 (*n* = 15)			
0 min	346.9 ± 148.5	302.3 ± 87.4	0.32
30 min	636.5 ± 112.8	640.7 ± 119.9	0.92
60 min	598.9 ± 135.0	629.9 ± 94.9	0.47
Group 3 (*n* = 15)			
0 min	348.9 ± 157.5	442.5 ± 198.4	0.02∗
30 min	540.9 ± 178.7	662.1 ± 167.8	0.01∗
60 min	611.7 ± 115.6	619.7 ± 166.3	0.84

All values given in nmol/L to convert to mg/dL are divided by 27.59.

∗Significant at *P* of ≤0.05.
